# Association of performance status, depression, and demographics with advance directive documentation in patients with glioblastoma

**DOI:** 10.1007/s00520-026-10492-6

**Published:** 2026-02-24

**Authors:** Heather Niccum Haag, Emily Lambrecht-Stock, Carlton G. Brown, Erica L. Dawson, Pierre Giglio, Loraine T. Sinnott, Laura Flora, Rory O’Malley, Elizabeth K. Arthur

**Affiliations:** 1 The Ohio State University Comprehensive Cancer Center – Arthur G. James Cancer Hospital and Richard J. Solove Research Institute, 460 W. 10thAve, Columbus, OH USA; 2 Zenith Healthcare Solutions, 1319 S. Calle Marcus, Palm Springs, CA 92264 USA; 3 Department of Neurology, The Ohio State University Wexner Medical Center, 460 W. 10th Ave, Columbus, OH USA; 4 Data Coordination and Analysis Center, The College of Optometry, The Ohio State University, 338 W 10th Ave, Columbus, OH USA

**Keywords:** Depression, Performance status, Advance care planning, Neuro-oncology, Glioblastoma

## Abstract

**Purpose:**

For those with a life-limiting illness, advance care planning (ACP) is essential for patient-centered care. However, there is limited research available on the timing and impact of ACP on patients with brain tumors, particularly with glioblastoma. The primary aim of this study was to determine if there is a relationship between advance directive (AD) documentation, a part of ACP, and depression, performance status scores, or demographics of patients with glioblastoma.

**Methods:**

The sample consisted of 146 patients with glioblastoma, 98 of whom had documented AD, defined as within the 14 days prior to diagnosis or during their disease course, at a single comprehensive cancer center. Demographic characteristics, depression scores, performance status scores, and AD documentation were extracted from electronic medical records over repeated clinical time points. Logistic regression, mixed model, and generalized estimating equations models were used to assess relationships between patient variables and documentation of AD.

**Results:**

No statistically significant difference in depression scores was found between those with and without documented AD. There was a statistically significant difference in performance status, such that those with worse performance status were more likely to have a documented AD. Patients who were female, older, and not married were more likely to have documented AD.

**Conclusion:**

In our sample, AD was more likely to be established for patients with advanced disease and specific demographic factors, whereas depression scores did not predict presence or absence of AD. This may indicate that patients with decreased performance status may be more receptive to completing AD than those with better performance status. Alternatively, it could reflect that providers are more likely to approach the subject as their health deteriorates. Patients preparing for neurosurgical intervention might be more receptive to AD completion and ACP discussion than patients in other phases of care. We offer recommendations to promote a more proactive approach to AD so that more patients are prepared by the time their performance status starts to decline.

**Supplementary information:**

The online version contains supplementary material available at 10.1007/s00520-026-10492-6.

## Background

Neuro-oncology is estimated to comprise 1.3% of all oncology cases and account for 3.1% of cancer-related deaths [1]. Glioblastoma is the most common and malignant central nervous system tumor, occurring between three and four people per 100,000 people annually [2]. Glioblastoma has poor survival outcomes, with median survival of 8 months in registry data and 14.6 to 21.1 months with standard therapy in clinical trials; although overall median survival remains under one year, there are comparatively better outcomes observed in younger adults [3, 4].

### Advance directives

Advance directives (AD), a part of advance care planning (ACP), can include documents such as a living will, a do-not-resuscitate (DNR) order, and a healthcare power of attorney (HCPOA). These documents are legally binding for end-of-life (EOL) care [5]. Several ACP studies identified that individuals are the least likely to have an AD if they are male, non-white, 18–34 years old, unpartnered, have less than a high school education, receive Medicaid, without chronic illness, and/or receive regular care [6, 7]. A systematic review of available literature for ACP in patients with glioblastoma (PWG) found rates of 4–55% for ACP documentation completion, with 29% having ACP completed within 6 months of diagnosis and only 55% having documents completed prior to death [8].

Given the guarded prognosis and potential for rapid functional decline with glioblastoma, ACP is a critical component of personalized care. Timely implementation of ACP in PWG is challenging, yet necessary, to match care received to care desired during the EOL phase [9]. For example, at the EOL, primary brain tumor patients have a higher frequency of confusion (50%) versus the general palliative population (14%) [10]. Over 50% of high-grade glioma patients have a diminished or absent decision-making capacity 4 months following their diagnosis [11]. The presence of anxiety and depression might prompt initial ACP engagement, decrease anxiety and depression related to EOL issues for patients and care-partners, and must occur before incapacity for autonomous decision-making [11–13]. Goals of care conversations and early palliative consultations are beneficial in guiding patients toward their EOL preferences including decreased hospital admissions at EOL, an increase in hospice utilization, and a greater chance of dying at the place of preference [14–17]. Barriers for providers initiating ACP discussions include lack of time, priority medical problems, and lack of continuity of care, while the ability to schedule ACP discussions is a facilitator [18]. Though 93% of clinicians viewed ACP to be moderately to extremely important, only 15% felt extremely confident in having ACP discussions due to perceived difficulty with ACP discussion frameworks, explaining ACP components, various cultural/religious backgrounds, or how to respond to patient/family emotions [18].

### Depression symptoms

Studies have shown PWG struggle with depression as a comorbidity. A prospective study of 86 PWG found that 90% had mild to moderate depression [19]. Another study confirming the validity of the Patient Health Questionnaire-9 (PHQ-9) scale for depressive symptoms in patients with cerebral gliomas found that 20% of the participants were diagnosed with major depressive disorder [20]. Long-term survivors of glioblastoma (greater than 2 years) describe an experience of detachment from others, leading to anxiety and depression [21]. Depression is commonly elevated among adults with advanced cancer, leading to the possible avoidance of difficult discussions around ACP or EOL [22]. Though women with glioblastoma experience more depression symptoms than men, when comparing men and women with glioblastoma with higher depression scores, men had worse survival outcomes [23]. A literature review and meta-analysis found that ACP interventions decrease decision conflict, depression, and anxiety [24]. Eight of the trials within the same meta-analysis found reduced depression symptoms among participants who engaged in ACP, with a standardized effect size of −1.2, though some variability likely exists among different populations [24]. These findings are relevant because people with brain tumors who endorse symptoms of depression have also been found to be more likely to think or talk about future medical care and were also more likely to complete legal forms compared with those who did not endorse depression symptoms [13].

### Performance status

PWG experience worse physical functioning than the general public, with one study showing declining Short Form Health Survey (SF-36) scores over time across most health domains, indicating worsening perceived health and physical functioning [25]. The Karnofsky Performance Status (KPS) scale indicates the impact of illness on functioning in oncology populations [26]. More specifically, it reflects a patient’s ability to complete activities of daily living independently and tolerate standard or aggressive cancer treatments. A cutoff score of 70 out of 100 (able to care for self, unable to work) is often used to identify their eligibility for clinical trials [26]. Higher KPS is associated with greater overall survival, including in recurrent glioblastoma [27–30]. Impaired functional status measured by a decreasing KPS has shown a direct link to increased chances of depression symptoms as measured by PHQ-9 in primary brain tumor patients [31]. Others have shown a KPS score of less than 70, tumor location, and postoperative complications are related to an increased risk of depression in PWG [32].

### Gaps in the literature

There is limited evidence describing the relationship between depression and performance status with documented AD in PWG. The lack of a meta-analysis for the relationship among depression, ACP, and cancer leads to the review of singular studies for research purposes [22]. This study aimed to assess the association between AD completion and depression, and performance status of PWG, hypothesizing that worsening in either of those factors would increase the likelihood of AD completion. The study team further hypothesized that demographic characteristics (age, gender, race, insurance status) would be associated with a greater likelihood of AD completion among PWG.

## Methods

### Study design and participants

The study team performed a secondary data analysis of medical record data from PWG treated at the study institution. The study protocol was approved by The Ohio State University Cancer Institutional Review Board (IRB) with a waiver of informed consent. This study was conducted in accordance with the Declaration of Helsinki.

### Sample

The sample was composed of PWG at a National Cancer Institute Designated Comprehensive Cancer Center. Medical records with a diagnosis date between January 1, 2016, and November 1, 2021, were screened for eligibility. Institutional IRB approval limited data extraction beyond the original submission in April 2023.

### Eligibility criteria

Eligible patients had pathologically confirmed glioblastoma, were ≥ 18 years old at diagnosis, had no documented incapacity to make their own medical decisions pre-diagnosis, received adjuvant therapy at the study institution, and had at least three medical visits at the study institution. Medical visits included hospitalizations and at least one outpatient neuro-oncology appointment. Patients were excluded if they had a diagnosis other than glioblastoma or completed an AD more than 14 days before their diagnosis.

### Procedures and data collection

#### Identification of subjects, and data collection

Medical record numbers, demographics, and depression scores were provided by the institution’s Honest Broker Oversight Committee. These records were screened for eligibility by the study team. Study data was documented securely using the Research Electronic Data Capture (REDCap®) database [33]. Data was extracted from a minimum of three and up to six clinical care trajectory time points as follows: initial consultation with the neuro-oncologist, following concurrent chemotherapy and radiation, following adjuvant chemotherapy, first disease progression (per MRI), hospice referral, and last follow-up. These timepoints represent critical phases of care versus defined elapsed time from diagnosis. Primary variables (i.e., depression, performance status, AD, demographic characteristics, and clinical information) were collected from the medical records and the provided dataset.

### Instruments

#### Advance directive documentation

Definitions of AD may vary by region. In this study, evidence of a patient having an AD included the documentation of a healthcare power of attorney (HCPOA), a living will (LW), and/or a do-not-resuscitate (DNR) order. Full code status alone was excluded as evidence of AD documentation due to being the institutional default code status with or without ACP discussions.

#### Depression

We measured depression using the PHQ-9, which contains nine questions that rank the frequency of symptoms and problems associated with depression in the preceding two weeks [34]. For patients with a total score of 0–2 on the first two questions (low depression symptoms) of the PHQ-9, questions 3–9 were not retrospectively recorded. These first two questions of the full PHQ-9 questionnaire are known as the PHQ-2 and capture the core features of depressed mood and anhedonia associated with a depressed state [35, 36]. Both the PHQ-2 and PHQ-9, when administered online, can provide good accuracy when measuring depression severity [36]. Variants of the PHQ-9, including the PHQ-2, function similarly when analyzing sensitivity and specificity [35]. PHQ-2 sensitivity and specificity are adequate for cutoff scores of 2 or greater [37]. Validity and reliability of the PHQ-9 have also been confirmed, with Cronbach’s *α* of 0.86–0.89 [34].

In this study, retrospective depression scores were provided as “PHQ-9 Total Score,” even in instances where only the PHQ-2 was performed. PHQ-9 scores range from 0 to 27; a score of 0 indicates no depressive symptoms. Missing PHQ data was extracted from narrative documentation by the registered nurse (RN) or physician. Documentation of patients having no depression symptoms was assigned a PHQ score of 0.

#### Performance status

Performance status was measured by the Karnofsky Performance Status (KPS) score within the physician note. The KPS reflects the rater’s evaluation of a patient’s functional status and can be used to infer an individual’s ability to tolerate treatment [38]. The KPS has documented strong interrater reliability (0.97) and validity, as well as predictive validity to estimate longevity in patients with terminal cancer [38]. While KPS can fluctuate throughout a patient’s course of treatment, KPS at hospital discharge at initial diagnosis is the most prognostic, regardless of the glioblastoma tumor location or size [39]. For physician notes with a documented KPS range (i.e., 50–60), the higher value was recorded.

For records without a KPS score, documentation of a negative review of systems by a registered nurse or physician in the medical record resulted in assignment of a score of 100. For records without a KPS score but with abnormal findings documented in the review of systems by a registered nurse or physician exam, the KPS score was coded as missing.

#### Data analysis

Sample characteristics were evaluated with descriptive statistics. To test the relationships between sample characteristics and AD documentation status, the study team used Pearson exact tests of the contingency tables for categorical variables and *t*-tests of mean difference for continuous variables. The *p* values for depression after cancer diagnosis are from chi-squared tests. The study team modeled the effect of AD status (independent variable) on depression status and KPS score (dependent variables). AD status was a 0/1 indicator of whether a patient had an AD at a given time. PHQ scores were converted to a dichotomous variable as the presence or absence of depression symptoms (PHQ = 0 vs PHQ = 1–18) due to the infrequency of scores greater than 0. Generalized estimating equations (GEE), generalizations of logistic regression that accommodate repeated measures over time, were used to model the dichotomous variable of depression [40]. For KPS, the study team fit a mixed model which is a generalization of linear regression that accommodates the correlation of within-subject repeated measures. Models controlled for time (in months), baseline age, and gender. The depression model also controlled for pre-existing depression and KPS. All analyses were performed using SAS version 9.4 for Windows. A level of 0.05 was used as the criterion for statistical significance.

## Results

### Sample characteristics

The sample consisted of 146 participants from a cohort of 301 potential cases, 67 of which were excluded for having AD greater than 14 days prior to surgery. Other exclusion reasons included pathology other than GBM; evidence of altered decision-making capacity (including incarceration); < 18 years of age; less than 3 protocol-defined encounters with neuro-oncologist; and no adjuvant therapy/adjuvant therapy ordered by an outside institution; ninety-eight participants had documented AD within the defined study window (Table 1). The mean baseline age was 58.3 years, with a range of 19.1 to 89.2 years. Most patients were white, married, and insured. There was an equal number of men and women.

**Table 1:** Clinical demographics

		AD documentation during study period (window start 14 days of surgery)		
	Overall *n* = 146	No *n *= 48	Yes *n* = 98	
	*N* (%)	*N* (%)	*N* (%)	*p* value
Gender				0.16
Male	73 (50%)	28 (58.3%)	45 (45.9%)	
Female	73 (50%)	20 (41.7%)	53 (54.1%)	
Race				0.89
Asian	3 (2.1%)	1 (2.1%)	2 (2.1%)	
Black or African American	11 (7.7%)	3 (6.4%)	8 (8.3%)	
White	129 (90.2%)	43 (91.5%)	86 (89.6%)	
Marital status				0.23
Single	32 (21.9%)	7 (14.6%)	25 (25.5%)	
Married	97 (66.4%)	38 (79.2%)	59 (60.2%)	
Divorced	7 (4.8%)	1 (2.1%)	6 (6.1%)	
Widowed	10 (6.8%)	2 (4.2%)	8 (8.2%)	
Insurance coverage				0.15
Medicare	56 (38.4%)	14 (29.2%)	42 (42.9%)	
Medicaid	12 (8.2%)	4 (8.3%)	8 (8.2%)	
Private insurance	57 (39%)	19 (39.6%)	38 (38.8%)	
Uninsured	21 (14.4%)	11 (22.9%)	10 (10.2%)	
Does participant have a power of attorney				** <.001**
No	65 (44.5%)	48 (100%)	17 (17.3%)	
Yes	81 (55.5%)		81 (82.7%)	
Does the participant have a DNR?				** <.001**
No	103 (70.5%)	48 (100%)	55 (56.1%)	
Yes	43 (29.5%)		43 (43.9%)	
Does the participant have a living will?				** <.001**
No	102 (69.9%)	48 (100%)	54 (55.1%)	
Yes	44 (30.1%)		44 (44.9%)	
	Mean (SD) Median (range)	Mean (SD) Median (Range)	Mean (SD) Median (Range)	*p* value
Age at diagnosis	58.3 (13.9) 61.5 (19.1 to 89.2)	55.1 (15.9) 60.3 (19.1 to 89.2)	59.8 (12.6) 62 (21.4 to 86.2)	**0.03**
Average KPS score *	75.1 (17) 80 (30–100)	79.0 (15.5)	70.2 (17.5)	** <.0001**
Presence of pre-existing depression				0.61
No	126 (86.3%)	40 (83.3%)	86 (87.8%)	
Yes	20 (13.7%)	8 (16.7%)	12 (12.2%)	
Depression symptoms post-diagnosis				0.12
No	130	40 (30.8%)	90 (69.2%)	
Yes	16	8 (50%)	8 (50%)	

^*^Average KPS score reflects the average of all repeated measures obtained during the study period for each respective group.

Bold entries indicate statistical significance

### Advance directives

AD documentation was defined as the presence of an HCPOA, DNR, or LW. The AD status of the 146 participants could change over time, but for 90 subjects (62%), this was not the case. Fifty-one had no AD throughout the follow-up period, while 39 participants had an AD at baseline, defined as up to 14 days before the date of diagnosis and up to the first appointment with the neuro-oncologist. In contrast, fifty-one participants had no AD throughout the follow-up period. Patients most often completed an AD between the 14 days before surgery and the first appointment with the neuro-oncologist, while others less often completed an AD after repeated encounters with their oncology team or not at all. Those participants with AD tended to be older, female, and single (Table 1). The 39 participants who had AD at baseline had created one within the 14 days before their surgery date up to the first appointment with the neuro-oncologist. This was the most common timing of AD documentation during the care trajectory. More participants with an AD document had an HCPOA (*n* = 81) compared to an LW (*n* = 44) or DNR (*n* = 43).

### Depression

Of all documented PHQ scores at all repeated participant timepoints, only 5.9% (*n* = 24) were greater than or equal to 1. 85.6% (*n* = 578) of PHQ scores were assigned a 0 according to the study’s missing data plan. These numbers reflect the few patients who had reported or assessed depressive symptoms documented by the PHQ and the degree of missing data.

Participants with pre-existing depression (*n* = 20) were more likely to experience depression during the care trajectory (*p* = 0.02). In a GEE model of depression with AD as the sole factor, the relationship between depression and AD completion was not statistically significant. Similarly, in a GEE model that included AD status, time, baseline age, gender, presence of pre-existing depression, and performance status, there was no evidence that having or not having an AD was significantly associated with depression status (Table 3).

### Performance status

Table 2 summarizes KPS statistics over time, with mean values representing the average KPS scores extracted for all patient cases at each clinical care trajectory time point. In a mixed model of performance status with ACP as the sole factor, the relationship of KPS with AD was statistically significant, with *p* ≤ 0.0001. In a mixed model that included AD status, time, baseline age, and gender, the association of AD status was *p* = 0.0001 (Table 3). All the factors (independent variables) had negative parameter estimates, indicating they were associated with lower KPS scores (i.e., worse performance status). Over time, KPS decreased, on average, 0.375 units with each passing month. Compared to when AD was not documented, KPS scores for those with AD documentation were, on average, 6.34 units less. Relative to males, the KPS scores of females were an average of 5.93 units less. The average KPS score throughout the care trajectory for those without AD was 79.0 and for those with AD was 70.2 (Table 1).

**Table 2:** Performance status scores and advance directive documentation

		Repeated measure					
	Overall	1	2	3	4	5	6
Overall events	*n* = 743	*n* = 146	*n* = 146	*n* = 146	*N* = 141	*N* = 118	*n* = 46
KPS score	75.1 (17) 80 (30 to 100) *n* = 572	80.5 (14.6) 80 (40 to 100) *n* = 132	80.2 (15) 80 (40 to 100) *n* = 116	77.3 (14.8) 80 (40 to 100) *n* = 112	74.6 (15.7) 75 (40 to 100) *n* = 108	63.7 (18) 60 (30 to 100) *n* = 78	52.3 (13.1) 50 (30 to 90) *n* = 26
AD documentation	*n* = 95	39	21	9	13	13	

A minimum of three and up to six clinical care trajectory time point appointments or hospitalizations with collected data were as follows: first appointment with the neuro-oncologist, appointment following concurrent chemotherapy and radiation, following adjuvant chemotherapy, first disease progression (per MRI), hospice referral, and last follow up

**Table 3:** Adjusted associations between patient characteristics and depression symptoms and KPS scores

Depression model			
	OR	95% CI	*p* value
Time relative to first appointment (months)	0.96	0.88–1.06	0.42
Has an AD (referent: no AD)	0.4	0.09–1.79	0.23
Age at first appointment (years)	1.01	0.95–1.07	0.85
Female (referent: male)	0.88	0.25–3.01	0.83
KPS score	0.96	0.92–0.99	**0.01**
Pre-existing depression (referent: no pre-existing depression)	3.91	1.22–12.55	**0.02**
KPS model			
	Slope	95% CI	*p* value﻿
Intercept	83.18	79.81–86.55	**<.01**
Time relative to first appointment (months)	–0.38	–0.5–0.25	**<.01**
Has an AD (referent: no AD)	–6.45	–9.62–3.28	**<.01**
Age at first appointment (years)	–0.18	–0.34–0.02	**0.03**
Female (referent: male)	–5.55	–9.8–1.3	**0.01**

Bold entries indicate statistical significance

## Discussion

This study retrospectively analyzed depression symptoms as measured by the total PHQ-9 score and performance status scores measured by the KPS in relation to AD completion in PWG. Demographics in this study most likely to have AD documentation were older, white, and female. Those with pre-existing depression before their diagnosis were most likely to experience depression symptoms during their care trajectory, though overall reporting of depression symptoms was low for further analysis. Those with lower KPS scores were more likely to have documented AD. Performance status and demographics results provide insight to key opportunities to introduce AD.

Though the study sample was relatively homogeneous, patients represented in this study are similar to those who might be diagnosed with glioblastoma in the geographical area. The Ohio Annual Cancer Report 2021 for the year 2018 reported 820 new cases of brain and CNS primary cancers among whites; 61 cases among black/African American Ohioans; and 12 Asian/Pacific Islander Ohioans; 381 males and 278 females [41]. Results from the current study indicate patients who are white, female, and older are more likely to have documented AD. This finding aligns with other studies showing that certain populations, such as women, those who are older, white individuals, and those with chronic diseases, are more likely to complete AD [7]. This suggests demographic factors may influence the likelihood of AD completion. Given these findings, there is opportunity to implement demographically tailored interventions. A systematic review of ACP in racial and ethnic minority groups found outcomes of ACP interventions were uncertain, but randomized control trials that included education had positive AD uptake and ACP engagement [42]. Understanding barriers helps patients document AD and improve care, and future studies should explore why ACP engagement varies by race, age, and sex.

There were two primary timepoints for AD among PWG—either during the initial surgical planning phase or when they experienced a decline in performance status. While some PWG wanted to complete ACP shortly after chemotherapy and radiation, a large majority preferred waiting until later in the disease trajectory [43]. Catching patients in these windows of opportunity for discussion is important and impactful for ACP.

The authors speculate the difference in AD type (HCPOA, LW, and/or DNR) may be the result of participant attitudes toward appointing a decision maker for “if” something happens to them versus “when” something happens and outlining the care desired once decisional incapacity occurs. A systematic review and meta-analysis of the stages of readiness for ACP found higher rates of signing a HCPOA than signing a LW in studies that reported by AD type, though DNR was excluded in this review [44]. The goal of ACP is to ensure the care a person desires is the care they receive. While ADs are a key tool in that process, ACP encompasses more than just completing an AD. Future qualitative studies should examine the reasons related to the timing of willingness to engage in ACP as well as rationale for completing some AD documents and not others. This is especially important for diagnoses, such as glioblastoma, that can lead to diminished decision-making capacity [10, 11].

There was no evidence found that having or not having an AD was significantly associated with depression status. While it was identified that the participants with pre-existing depression were more likely to experience depression during the care trajectory, the overall observed incidence of depression was much lower than what is known to be present in the oncology population. Depression impacts approximately 1 in 4 patients with cancer, with self-report measures of depression symptoms ranging from 5% to greater than 40% [45]. Lack of historical documentation of depression, potentially reflecting limitations in prior assessment practices, hindered study measurement in those without documented pre-existing depression. A prospective study with more control over collected data may better determine if ACP, the greater umbrella of AD, could be utilized as a tool to navigate the most difficult of conversations and alleviate depression in PWG.

For performance status, there was a statistically significant difference in KPS scores for those who had and did not have a documented AD. Those with a lower KPS score, or worse performance status, were more likely to document AD. At the time of literature review for this research, there were no previous studies found that explored the correlation between performance status and AD completion or ACP engagement. This study’s finding may be a result of the clinician’s comfort with goals of care discussion with those with decreased performance status, or it could be that patients with better overall health are more likely to resist recommendations to establish AD. The dearth of available literature exploring the correlation between performance status and ACP highlights the need for future studies.

## Limitations

Study limitations are important to acknowledge when examining the generalizability of study results. Historical data is not collected for the purpose of research and thus may not provide the needed information to answer research questions. PHQ-2 and PHQ-9 scales, although available, were not historically documented consistently. Overall, the sample was relatively homogenous (Table 1). Missing data limits this study’s conclusion on the correlation of AD and depression. Results may not be generalizable to racial minorities. As sexual orientation and gender identity data, social determinants of health, and social support are becoming more frequently assessed, future studies can explore these relationships with ACP and AD documentation. The criteria for diagnosing GBM have changed since the data collection window. Clinical features beyond adult age and pathologic diagnosis of GBM were not included in the analysis (extent of resection, treatment type, molecular characteristics).

## Conclusion

For those with a life-limiting illness, ACP is essential for patient-centered care. Although those who are women, older, and single are more likely than their counterparts to document an AD, screening for ACP and initiating discussions should occur with all patient demographics. Further qualitative research could attempt to tease out why PWG or other primary brain tumors are more likely to engage in ACP and complete AD. Validated measures for symptoms, such as depression, should be embedded in clinician workflows for every patient, every visit, with best practice alerts for referrals to support services and/or palliative care. Results of this study suggest a decrease in performance status is another opportunity to introduce ACP for those who do not have AD. It is important to offer ACP to those who also have good performance status. Clinicians should assess their own attitudes about ACP to help healthcare providers engage more effectively in discussions with patients. The results contribute to the limited research available on the timing of AD completion and declining performance status in PWG. The multidisciplinary team plays a critical role in advocating for ACP, assessing mental health needs, and supporting PWG and their families through difficult conversations.

## Supplementary information

Below is the link to the electronic supplementary material.

ESM 1 (DOCX 16.4 KB)

## Data Availability

The data that support the findings of this study are available from the authors. Restrictions apply to the availability of these data and are not publicly available. Data are subject to all confidentiality and HIPAA requirements. Permissions to access data are required from the Office of Responsible Research Practices at The Ohio State University.
