# Supplementary material for: Association of performance status, depression, and demographics with advance directive documentation in patients with glioblastoma
Source: Support Care Cancer. 2026 Feb 24;34(3):245. doi: 10.1007/s00520-026-10492-6 (PMC12932399; doi:10.1007/s00520-026-10492-6)
Supplement: Supplementary file 1 — (DOCX 16.4 KB) [file 520_2026_10492_MOESM1_ESM.docx]

Supplemental Table:

|  | | Repeated measure | | | | | |
| --- | --- | --- | --- | --- | --- | --- | --- |
|  | Overall | 1 | 2 | 3 | 4 | 5 | 6 |
| Overall Events | n=743 | n=146 | n=146 | n=146 | N=141 | N=118 | n=46 |
| **PHQ-9 Total Score** | n=675 |  |  |  |  |  |  |
| 0 | 651 (96.4%) | 127 (96.2%) | 129 (97%) | 128 (97.7%) | 129 (97.7%) | 100 (94.3%) | 38 (92.7%) |
| 1 | 8 (1.2%) | 2 (1.5%) | 2 (1.5%) | 1 (0.8%) | 1 (0.8%) | 1 (0.9%) | 1 (2.4%) |
| 2 | 6 (0.9%) | 1 (0.8%) |  | 2 (1.5%) | 1 (0.8%) | 1 (0.9%) | 1 (2.4%) |
| 3 | 1 (0.1%) |  | 1 (0.8%) |  |  |  |  |
| 4 | 4 (0.6%) | 1 (0.8%) | 1 (0.8%) |  |  | 1 (0.9%) | 1 (2.4%) |
| 7 | 2 (0.3%) |  |  |  | 1 (0.8%) | 1 (0.9%) |  |
| 14 | 1 (0.1%) | 1 (0.8%) |  |  |  |  |  |
| 15 | 1 (0.1%) |  |  |  |  | 1 (0.9%) |  |
| 18 | 1 (0.1%) |  |  |  |  | 1 (0.9%) |  |

A minimum of three and up to six clinical care trajectory time point appointments or hospitalizations with collected data were as follows: first appointment with the neuro-oncologist, appointment following concurrent chemotherapy and radiation, following adjuvant chemotherapy, first disease progression (per MRI), hospice referral, and last follow up.
